# Investigating esophageal sarcomatoid carcinoma and its comparison with esophageal squamous cell carcinoma on clinicopathological characteristics, prognosis, and radiomics features: a retrospective study

**DOI:** 10.3389/fonc.2024.1398982

**Published:** 2024-07-01

**Authors:** Binbin Hu, Kejia Zhao, Yushang Yang, Yi Zhang, Guihong Liu, Haiyan Zeng, Bingwen Zou

**Affiliations:** ^1^ Department of Radiation Oncology, Division of Thoracic Oncology, Cancer Center, West China Hospital, Sichuan University, Chengdu, Sichuan, China; ^2^ Department of Thoracic Surgery and Institute of Thoracic Oncology, West China Hospital, Sichuan University, Chengdu, Sichuan, China; ^3^ Western China Collaborative Innovation Center for Early Diagnosis and Multidisciplinary Therapy of Lung Cancer, Chengdu, Sichuan, China; ^4^ Research Core Facility of West China Hospital, Sichuan University, Chengdu, Sichuan, China

**Keywords:** esophageal sarcomatoid carcinoma, esophageal squamous cell carcinoma, clinicopathological characteristics, prognosis, radiomics features

## Abstract

**Introduction:**

Esophageal sarcomatoid carcinoma (ESC) is a rare pathological subtype of esophageal carcinomas, wherein its epithelial component typically demonstrates squamous cell carcinoma (SCC). However, the clinicopathological features and prognosis of ESC remain unclear, alongside its unique aspects compared to esophageal SCC (ESCC).

**Methods:**

Between January 2008 and December 2018, we retrospectively reviewed 67 ESC patients treated at West China Hospital. Among them, 51 patients with resected ESC were matched with 98 resected ESCC patients over the same period using propensity score matching at 1:2. The survival time and radiomics features of the two groups were compared.

**Results:**

A total of 59 patients with resected ESC and eight patients with non-resected ESC were enrolled. Progression-free survival (PFS) and overall survival (OS) were significantly different in patients with different TNM stages (*p* < 0.001). A multivariate analysis showed that length of tumor was an independent factor for OS in resetable ESC (*p* = 0.041). Among matched ESC and ESCC patients, OS was significantly longer for patients with ESC than those with ESCC (5-year OS, 61.1% vs. 43.6%; HR 0.59, 95% CI 0.35–0.96; *p* = 0.032). A Rad-score for discriminating ESC from ESCC containing two CT-derived radiomics features was developed [area under the curve: 0.823 (95% CI 0.732–0.913) in the training cohort and 0.828 (95% CI 0.636–1.000) in the validation cohort, respectively].

**Conclusions:**

ESC has a better prognosis when compared with ESCC. By developing a radiomics prediction model, we provide reliability and convenience for the differential diagnosis of ESC from ESCC.

## 1 Introduction

Esophageal sarcomatoid carcinoma (ESC) is a rare malignant tumor, representing approximately 0.5%–2.8% of all esophageal carcinomas (1). Carcinomatous and sarcomatous components coexist in this malignancy with an uncertain histogenesis. Based on this, multiple designations such as carcinosarcoma, spindle-cell squamous cell carcinoma, polypoid cancer, pseudosarcoma, and pseudosarcomatous carcinoma have been assigned to this neoplastic disorder (2). Despite variations in nomenclature among WHO systems, an increasingly precise delineation has emerged recently: if clear heterogenous sarcoma components exist within sarcomatoid interstitia (e.g., leiomyosarcoma, chondrosarcoma), it is carcinosarcoma; otherwise, it is sarcomatoid carcinoma (3).

The carcinomatous component of ESC is usually squamous cell carcinoma (SCC) (4). Lacking an established standard for treatment, ESC is empirically managed following the standards of esophageal SCC (ESCC) (5–7). Few comprehensive assessments have elucidated ESC and ESCC variances, but still controversy exists regarding the prognosis of ESC and ESCC, underscoring their distinct treatment requirements (8–10). What is more, diagnosing ESC can occasionally mimic ESCC, particularly in puncture biopsy. It is noteworthy that ESC presents as a large polypoid mass growing into the lumen in most cases, making patients symptomatic early in the disease course (4). The typical morphological characteristics of ESC seem to be able to help doctors to distinguish it with ESCC in CT images. Radiomics, offering high-throughput medical image data with the advantages of features such as real time, objective, noninvasive, and reusability, may facilitate differential diagnosis (11, 12).

In this study, we retrospectively reviewed ESC patients to perform a comprehensive analysis of their clinicopathological features and prognosis. After using propensity score matching (PSM), we further compared the prognosis and radiomics features between ESC and ESCC patients to construct a prediction model, contributing to a deeper understanding of these two esophageal carcinoma subtypes.

## 2 Patients and methods

### 2.1 Patient selection and data collection

**Figure 1:**
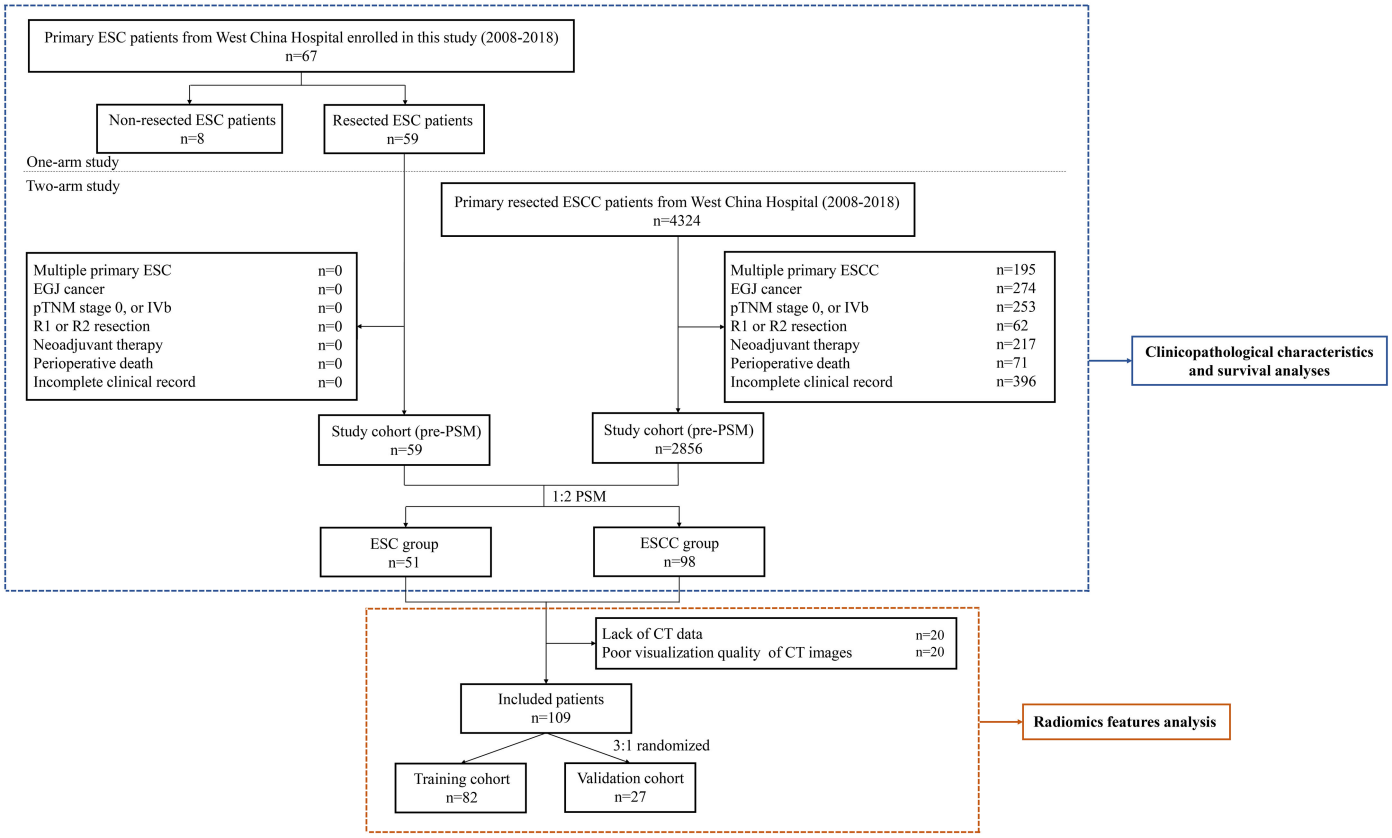
Flow chart of the study. ESC, esophageal sarcomatoid carcinoma; ESCC, esophageal squamous cell carcinoma; EGJ, esophagogastric junction; pTNM, pathological TNM; PSM, propensity score matching.

This retrospective study consisted of a one-arm and a two-arm analyses. A total of 67 ESC patients treated at West China Hospital between January 2008 and December 2018 were enrolled in the one-arm analysis (**Figure 1**). Baseline data including age, sex, smoking history, alcohol history, pathological component, tumor location, length of tumor, histological grade, TNM stage, and treatment information were collected. TNM stage was determined according to the 8th edition of the American Joint Committee on Cancer (AJCC) esophageal and esophagogastric junction (EGJ) cancer staging system. All diagnoses of ESC underwent rigorous reconfirmation using morphological characteristics and immunophenotypic staining criteria, evaluated by two highly experienced pathologists adhering strictly to the World Health Organization classification criteria.

Of these patients, 59 ESC patients who received esophagectomy with extensive lymphadenectomy were reviewed for survival comparison with resected ESCC patients at West China Hospital over the same period in the two-arm analysis. The exclusion criteria included patients with multiple primary tumors or EGJ cancer, patients with pathological TNM (pTNM) stage 0 or IVb, patients who received incomplete resection (R1 and R2 resection) or neoadjuvant therapy, patients who died during the perioperative period, and patients with an incomplete clinical record. In addition, a PSM method was used to minimize the impact of confounding factors (13). The propensity score for every patient was calculated with a logistic regression model, including the following variables: age, sex, tumor location, histological grade, pathological T (pT) stage, pathological N (pN) stage, and pTNM stage. After performing a 1:2 matching protocol with a caliper width of 0.2, 51 ESC patients were matched with 98 ESCC patients in this analysis (**Figure 1**).

Additionally, a radiomics feature analysis from contrast-enhanced chest CT images obtained upon initial diagnosis was performed in the matched ESC and ESCC groups. After excluding 20 patients with incomplete CT data and 20 with subpar image quality, a 3:1 random split yielded 82 patients in the training cohort and 27 patients in the validation cohort (**Figure 1**). This study was approved by the Institutional Review Board (IRB) of West China Hospital (no. 2021–767). Informed consent was waived for this research.

### 2.2 Follow-up and assessment

Routine follow-ups were scheduled at intervals of 3 months during the initial 2 years, progressing to semi-annually over three consecutive years and eventually yearly thereafter. Each follow-up session included a physical examination, laboratory tests, contrast-enhanced CT, and X-ray barium meal radiography or/and endoscopy. Overall survival (OS) was calculated from inception of treatment until death resulting from any underlying cause or the attainment of last follow-up. Progression-free survival (PFS) was measured from the initial day of treatment to the day of disease progression or mortality from any cause.

### 2.3 CT imaging

Each subject underwent imaging on Philips Brilliance 64-slice detector-row machines (Philips Healthcare, Cleveland, OH, USA), with specific settings adopted: tube potential at 120 kVp, current intensity ranging from 200 to 250 mA, rotation time between 0.5 and 1 s, pitch lying in the range of 0.891 to 1.235; collimation conforms to 64 × 0.625 mm, field of view (FOV) extending from 400 to 500 mm, matrix size set at 512 × 512, layer thickness of 5 mm, and layer spacing of 5 mm. Intravenous delivery of nonionized contrast medium (1.5–2.0 mL/kg, iohexol: Beijing Beilu Pharmaceutical, Beijing, China) was implemented through the antecubital vein, using a power injector (Stellant D, Medrad, Indianola, PA, USA) with a rate of 2 to 3 mL/s. All examinations were taken pre- and post-contrast administration. All contrast-enhanced CTs adopted the arterial phase for the image segmentation and radiomics elaboration.

### 2.4 ROI segmentation and radiomics feature extraction

**Figure 2:**
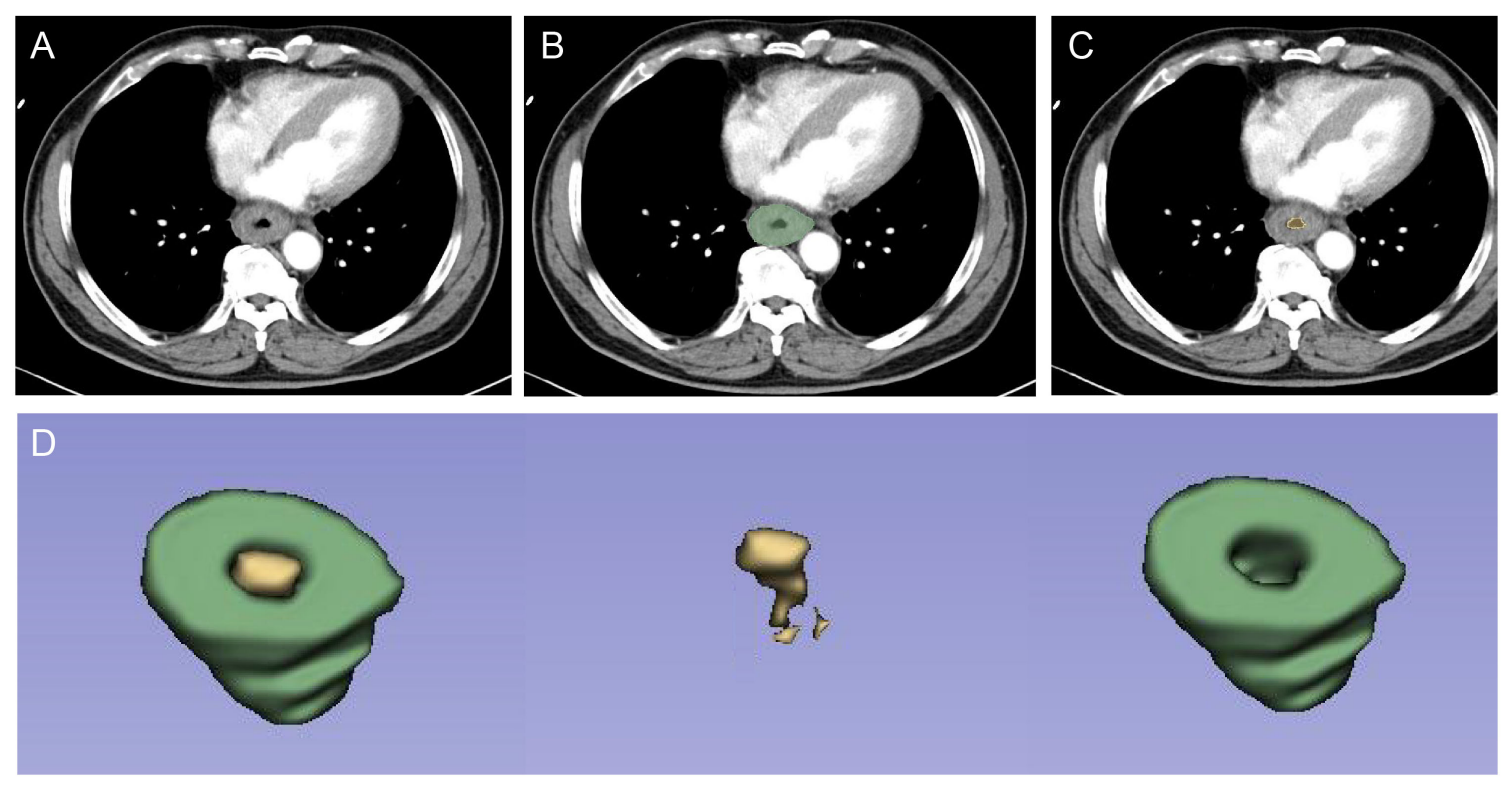
ROI segmentation for radiomic analysis. First, radiologists checked the lesion on contrast-enhanced CT at the arterial phase **(A)**. Then, the GTV **(B)** and intra-luminal air from GTV **(C)** were drawn, respectively. Finally, the computer automatically segmented the two regions, which resulted in the identification of the volumetric features of the tumor (ROI, green) and intra-luminal air (yellow) **(D)**. ROI was used for subsequent radiomics feature extraction and model construction. ROI, region of interest; GTV, gross tumor volume.

Pretreatment contrast-enhanced CT images were procured for each patient. The primary tumor volume (GTV) was defined as region of interest (ROI), drawn using 3D slicer V5.0.2 (https://www.slicer.org/). All pixels exhibiting attenuation levels below -50 HU were excluded, effectively eliminating intra-luminal air from definitive GTVs (**Figure 2**). “Pyradiomics package” plug-in was used to extract radiomics features. A total of 107 original features and 744 wavelet features, including shape-based histogram features, first-order statistics features (IH, intensity histogram), and texture features (gray-level co-occurrence matrix, GLCM; gray-level dependence matrix, GLDM; gray-level run-length matrix, GLRLM; gray-level size-zone matrix, GLSZM; and neighboring gray-tone difference matrix, NGTDM) were collected per ROI.

### 2.5 Radiomics model construction

Initially, we discarded two parameters exhibiting intraclass correlation coefficients (ICCs) below 0.8 and performed the least absolute shrinkage and selection operator (LASSO) analysis to discern the features correlated with esophageal cancer histology in the training cohort. Optimal lambda (λ) was selected from the LASSO model utilizing 10-fold cross-validation, adhering to minimal criteria. Then, we conducted a multivariate logistic regression analysis using likelihood ratio in both stepwise selection to select the most predictable features and construct the model. For each patient, a radiomics score (Rad-score) was calculated through a linear combination of selected features that had their weights dictated by corresponding coefficients.

### 2.6 Statistical analysis

Categorical variables were presented as numbers (%) and analyzed using chi-square tests, while continuous variables were expressed as mean ± standard deviation (SD) or median ± range and compared using one-way ANOVA. Survival curves were estimated by using the Kaplan–Meier method and compared using the log-rank test. Univariate and multivariate Cox proportional hazards regression, respectively, were used to evaluate the prognostic factors and to calculate hazard ratios (HRs) and 95% confidence interval (CI) for OS. Only variables demonstrating statistical discernment during univariate evaluations were incorporated into multivariable regressions. Subgroup analyses of OS were also performed by using the Kaplan–Meier method and the log-rank test. Two-sided *p* < 0.05 was considered statistically significant.

We used “irr package” to perform ICC analysis and “glmnet package” to perform the LASSO regression analysis. Model evaluation was assessed by the ROC curve analysis using “pROC package”, and the area under the curve (AUC) with 95% CI, sensitivity, and specificity were calculated. Calibration curve using “rms package” was also plotted to assess the calibration of the model. All statistical analyses were performed using SPSS version 22.0 and R statistical software (version 4.1.0).

## 3 Results

### 3.1 Clinical characteristics

A total of 67 ESC patients, including 59 resected patients and eight non-resected patients, were enrolled in our study. The clinical characteristics of these two populations are summarized in **Table 1**; **Supplementary Table S1**, respectively. Among patients with resected ESC, the median age was 63 years (range, 45 to 84 years). Patients with upper thoracic, middle thoracic, and lower thoracic ESC accounted for 8.5% (5/59), 69.5% (41/59), and 22.0% (13/59) of the entire cohort, respectively. All of the patients underwent esophagectomy with extensive lymphadenectomy. The pTNM stage distribution was stage I in 16 (27.1%) cases, stage II in 28 (47.5%) cases, stage III in 15 (23.7%) cases, and stage IV in one (1.7%) case. A total of 16 (27.1%) patients received adjuvant treatment, including three cases of chemotherapy, four cases of radiation, and nine cases of chemoradiotherapy.

**Table 1:** Clinical characteristics of patients with resected ESC.

Variables	Overall (*n* = 59)	Variables	Overall (*n* = 59)
Age (mean ± SD)	62.4 ± 8.6	Surgical technique (%)	
Age, years (%)		McKeown	13 (22.1)
≤60	25 (42.4)	Ivor-Lewis	12 (20.3)
>60	34 (57.6)	Sweet	34 (57.6)
Gender (%)		Histological grade (%)	
Male	51 (86.4)	G1	1 (1.7)
Female	8 (13.6)	G2	3 (5.1)
Tobacco (%)		G3	34 (57.6)
Yes	40 (67.8)	Gx	21 (35.6)
No	19 (32.2)	pT stage (%)	
Alcohol (%)		T1	17 (28.8)
Yes	38 (64.4)	T2	16 (27.1)
No	21 (35.6)	T3	21 (35.6)
Tumor location (%)		T4	5 (8.5)
Upper thoracic	5 (8.5)	pN stage (%)	
Middle thoracic	41 (69.5)	N0	38 (64.4)
Lower thoracic	13 (22.0)	N1	12 (20.3)
Length of tumor (mean ± SD)	4.9 ± 2.4	N2	8 (13.6)
Length of tumor, cm (%)		N3	1 (1.7)
≤3	19 (32.2)	pTNM stage (%)	
>3	40 (67.8)	I	16 (27.1)
No. of dissected nodes (mean ± SD)	17.7 ± 9.3	II	28 (47.5)
No. of dissected nodes (%)		III	14 (23.7)
≤10	14 (23.7)	IV	1 (1.7)
>10	45 (76.3)	Adjuvant treatment	
LND approach (%)		Yes	16 (27.1)
2-field	40 (67.8)	No	43 (72.9)
3-field	19 (32.2)		

ESC, esophageal sarcomatoid carcinoma; SD, standard deviation; LND, lymph node dissection; pT, pathological T; pN, pathological N; pTNM, pathological TNM.

In the cohort of patients with non-resected ESC, all of them were male, and the median age was 63 years (range, 43 to 82 years). Patients with upper thoracic, middle thoracic, and lower thoracic ESC accounted for 25.0% (2/8), 50.0% (4/8), and 25.0% (2/8) of the entire cohort, respectively. All of the patients had distant metastasis, and six patients (75.0%) received anti-tumor therapy, including one case of palliative surgery, three cases of chemoradiotherapy, and two cases of chemotherapy.

### 3.2 Pathological features

All patients in our study underwent endoscopic biopsy before surgery or other treatments. Only 37.3% (25/67) of patients had a clear pathological diagnosis of sarcomatoid carcinoma through this way. The proportion of carcinomatous and sarcomatous components may affect the patients’ needle biopsy results to some extent. A 67-year-old male patient with a mass of SCC components admix with few sarcomatous components was initially diagnosed as ESCC, while another 63-year-old male patient with a large amount of sarcomatous component and a small amount of SCC component was accurately diagnosed as ESC through endoscopic biopsy (**Supplementary Figure S1**). The predominant component was carcinoma in 20 (29.9%) patients, sarcomatoid carcinoma in 11 (16.4%) patients, and unknown in 36 (53.7%) patients in our study. In terms of carcinomatous, SCC was the most common histological type (95.5%, 64/67) in this cohort, and others included two cases of basaloid squamous carcinoma and one case of adenosquamous carcinoma. Immunohistochemical staining was performed in 63 cases, showing cytokeratin (CK) (45/52), epithelial membrane antigen (EMA) (31/42), vimentin (Vim) (32/35), smooth muscle antigen (SMA) (11/34), and S-100 (7/40) as positive to a certain degree.

### 3.3 Patient outcomes

**Figure 3:**
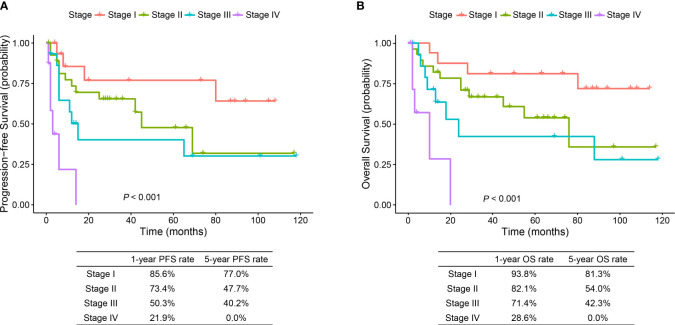
Progression-free survival and overall survival curves of stage I–IV ESC patients. ESC, esophageal sarcomatoid carcinoma; PFS, progression-free survival; OS, overall survival.

The median follow-up time was 29.0 months (range, 1.0 to 118.0 months). PFS and OS were significantly different in patients with different TNM stages (*p* < 0.001) (**Figure 3**). The 1- and 5-year OS rates were 93.8% and 81.3% in stage I patients, 82.1% and 54.0% in stage II patients, 71.4% and 42.3% in stage III patients, and 28.6% and 0.0% in stage IV patients, respectively. Predictors of OS in patients with resected ESC were analyzed such that length of tumor (*p* = 0.044), pT stage (*p* = 0.047), and pTNM stage (*p* = 0.048) were significant factors in the univariate analysis. Multivariate analysis showed that length of tumor was an independent factor for OS (*p* = 0.041) (**Table 2**).

**Table 2:** Univariable and multivariable Cox regression analyses for predictors of overall survival in patients with resectable ESC.

Variables	Univariable analysis			Multivariable analysis		
	HR	95% CI	*p* value	HR	95% CI	*p* value
Age, years			0.061			
≤60	Ref					
>60	2.42	0.96 to 6.11				
Gender			0.140			
Male	Ref					
Female	0.22	0.03 to 1.64				
Tobacco			0.309			
Yes	Ref					
No	0.62	0.25 to 1.56				
Alcohol			0.706			
Yes	Ref					
No	0.85	0.36 to 1.99				
Tumor location			0.298			
Upper thoracic	Ref					
Middle thoracic	3.01	0.40 to 22.89	0.288			
Lower thoracic	4.70	0.57 to 38.45	0.149			
Length of tumor, cm			0.044			0.041
≤3	Ref			Ref		
>3	3.02	1.03 to 8.87		3.10	1.05 to 9.18	
No. of dissected nodes			0.124			
≤10	Ref					
>10	0.50	0.20 to 1.21				
LND approach			0.425			
2-field	Ref	0.27 to 1.73				
3-field	0.69					
Surgical technique			0.174			
McKeown	Ref					
Ivor-Lewis	4.46	0.92 to 21.54	0.063			
Sweet	32.96	0.67 to 13.08	0.152			
Histological grade			0.286			
G1	Ref					
G2	0.09	0.01 to 1.51	0.094			
G3	0.13	0.02 to 1.11	0.063			
Gx	0.13	0.02 to 1.15	0.067			
pT stage			0.047			0.263
T1/T2	Ref			Ref		
T3/T4	2.30	1.01 to 5.23		1.63	0.69 to 3.86	
pN stage			0.089			
N0	Ref					
N1–3	2.02	0.90 to 4.52				
pTNM stage			0.048			0.075
I	Ref			Ref		
II–IV	3.00	1.01 to 8.94		2.80	0.90 to 8.67	
Dominant component			0.519			
Carcinomatous	Ref					
Sarcomatoid	0.574	0.16 to 2.10	0.402			
Unknown	0.640	0.27 to 1.51	0.307			
Adjuvant treatment			0.751			
Yes	Ref					
No	1.16	0.46 to 2.93				

ESC, esophageal sarcomatoid carcinoma; HR, hazard ratio; CI, confidence interval; Ref, reference; LND, lymph node dissection; pT, pathological T; pN, pathological N; pTNM, pathological TNM.

### 3.4 Survival comparison between the matched ESC and ESCC groups

**Figure 4:**
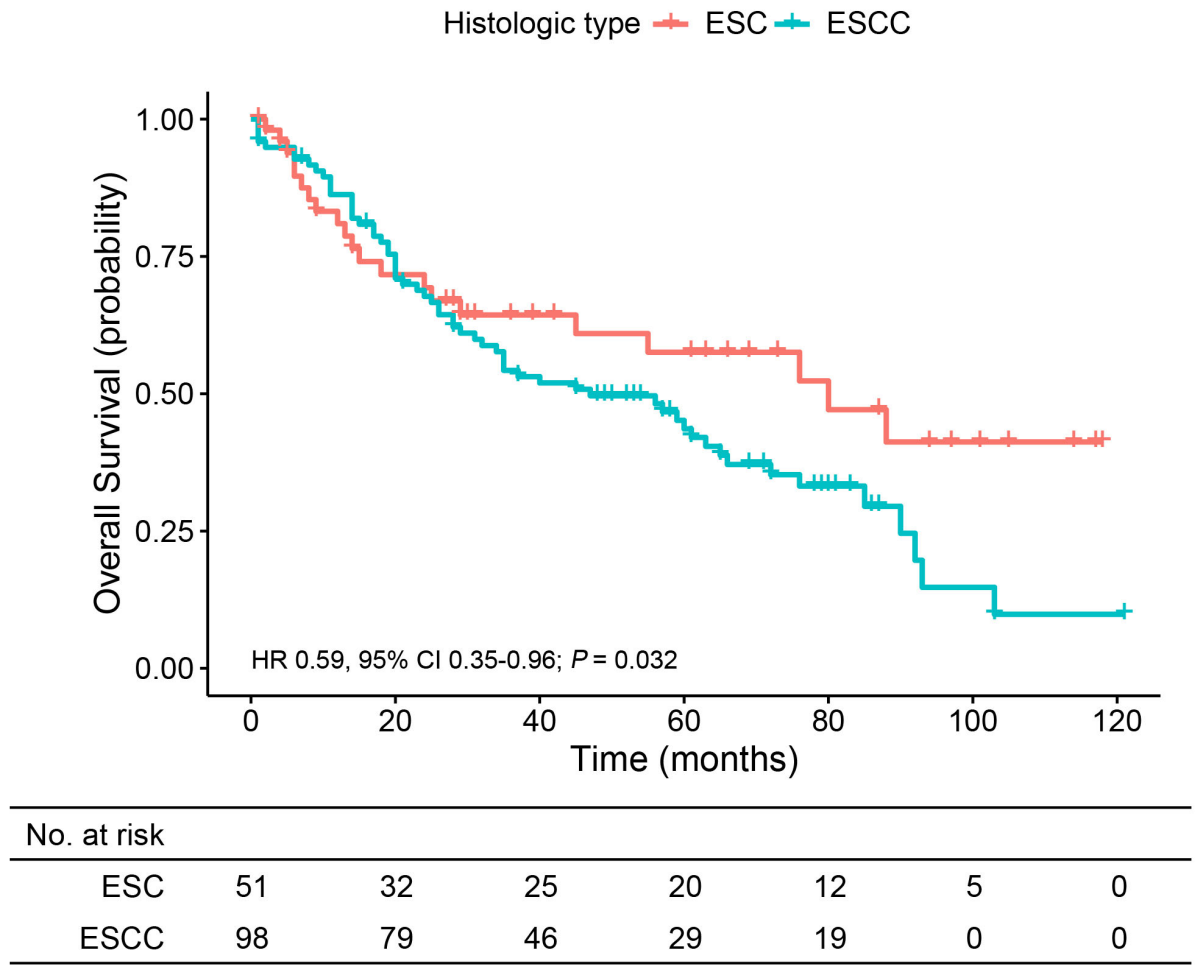
Overall survival analysis of patients from the matched ESC and ESCC groups. ESC, esophageal sarcomatoid carcinoma; ESCC, esophageal squamous cell carcinoma.

To further understand the prognosis of patients with ESC, we compared the OS of ESC patients with those with ESCC, one of the common histological types in esophageal cancer. The baseline characteristics of resected ESC and ESCC patients before and after PSM are summarized in **Supplementary Table S2**. After PSM, each variable in the two groups became more balanced. The median follow-up time was 39.0 months (range, 2.0 to 118.0 months) in the ESC group and 35.0 months (range, 1.0 to 125.0 months) in the ESCC group. OS was significantly longer for ESC patients than ESCC patients (5-year OS, 61.1% vs. 43.6%; HR 0.59, 95% CI 0.35–0.96; *p* = 0.032) (**Figure 4**). Additionally, the subgroup analyses for patients with age ≤60 years (HR 0.35, 95% CI 0.12–1.02; *p* = 0.045), patients with upper thoracic tumor (HR 0.15, 95% CI 0.02–1.31; *p* = 0.048), and patients with poorly differentiated or undifferentiated tumor (HR 0.51, 95% CI 0.27–0.96; *p* = 0.032) indicated better survival outcome in the ESC group.

### 3.5 Radiomics feature analysis for discriminating ESC from ESCC

**Figure 5:**
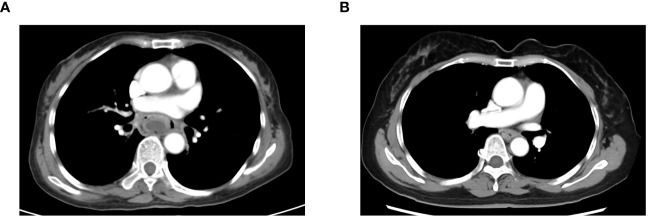
Representative contrast-enhanced chest CT images of the ESC and ESCC patients. **(A)** A CT image of a 57-year-old woman with pathological stage II ESC on the middle thoracic location showed that the tumor presented as a large lump growing into the lumen with an unclear boundary, and the peripheral lesion had a moderate enhancement. **(B)** A CT image of a 63-year-old man with pathological stage III ESCC on the middle thoracic location showed that the tumor presented as a thickening of the esophagus wall with an even enhancement. Both CT images were taken at the arterial phase. ESC, esophageal sarcomatoid carcinoma; ESCC, esophageal squamous cell carcinoma.

The CT features of tumor lesions in ESC and ESCC patients were different. **Figure 5** displays typical contrast-enhanced chest CT images for ESC and ESCC individuals respectively. Unlike ESCC, distinguished by an expanding annular wall and intensified wall thickness and initially undetectable due to its minute size, ESC manifests at initial stages as a bulky mass expanding into the lumen with a distinct boundary imparting an eccentric crescent or crevice appearance. Notably, an ESC lesion exhibits a moderate peripheral enhancement and lower density compared to ESCC.

Given these differences, we further performed a radiomics feature analysis. Patients with ESC or ESCC were divided into the training cohort (*n* = 82) and validation cohort (*n* = 27). There were no significant differences in patient characteristics between these two cohorts (**Supplementary Table S3**). LASSO logistic regression was employed to reduce the dimensionality of the extracted radiomics features and screen out the optimal radiomics features in the training cohort (**Supplementary Figures 2A, B**). As a result, two radiomic features were finally screened out, and the Rad-score was calculated as follows: Rad-score=-30.035152+4.309571*original_gldm_DependenceEntropy-18.261999*wavelet_LLL_glcm_InformationalMeausureofCorrelation1.

**Figure 6:**
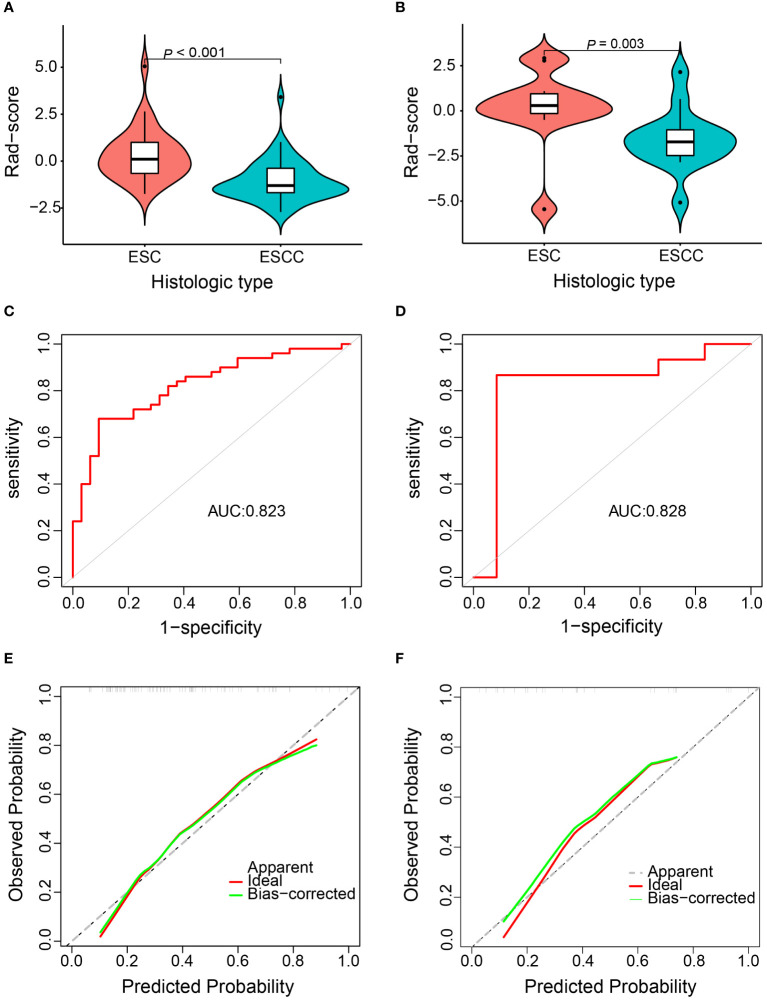
Diagnostic efficiency assessment of the radiomics model for discriminating esophageal sarcomatoid carcinoma (ESC) from esophageal squamous cell carcinoma (ESCC). Distribution of Rad-score between the ESC and ESCC patients in the training cohort **(A)** and validation cohort **(B)**. Receiver operation characteristic curve in the training cohort **(C)** and validation cohort **(D)**. Calibration curve in the training cohort **(E)** and validation cohort **(F)**.

The Rad-score showed a significant difference between ESC and ESCC patients in both the training (*p* < 0.001) and validation cohorts (*p* = 0.003) (**Figures 6A, B**). The AUCs of the Rad-score predicted ESC histological type in the training and validation cohort were 0.823 (95% CI 0.732–0.913) and 0.828 (95% CI 0.636–1.000), respectively (**Figures 6C, D**). The optimum cutoff of Rad-score generated by the AUC in the training cohort was -0.943. At this cut-off, sensitivity and specificity equaled 90.6% and 68.0% in the training cohort and 91.7% and 73.3% in the validation cohort, respectively. The calibration curve of this prediction model demonstrated a good agreement between prediction and observation in both the training and validation cohorts (**Figures 6E, F**).

## 4 Discussion

Given the rare incidence of ESC, little is known about its clinicopathological characteristics, radiomics features, and prognosis. Most of the published literature mainly consists of reports. In this study, we retrospectively reviewed 67 patients with ESC, one of the largest series of this rare malignancy, and performed a comparative study of ESC and ESCC, which provided a systematic evaluation of ESC.

The current literature employs diverse terminologies to refer to ESC, reflecting the uncertain pathogenesis of this malignancy. None of the reported theories can fully rationalize the pathological peculiarities of sarcomatoid carcinoma seen under the microscope such that these tumors are biphasic with a mixture of carcinoma and malignant sarcomatoid elements (4, 14, 15). Though ESC is traditionally believed to predominantly feature sarcomatoid components, a substantial portion of those with a clear mix of carcinomatous and sarcomatoid elements in our study exhibited SCC dominance. Chino and colleagues proposed that which component dominates may be related to the gross types of ESC (16). They found that the majority of the protruding ESC consisted of the sarcomatous component, while the ulcerating ESC mainly consisted of SCC. However, unlike our expected results, there was no significant difference in survival prognosis among patients with diverse proportions of carcinomatous and sarcomatoid elements. This may be due to the statistical bias caused by a lack of relevant pathological data in 53.7% of patients. Nonetheless, further investigation into the histogenesis and proportion of these two components is warranted, which may offer counsel for therapy in ESC patients.

Currently, there are no guidelines for the standard treatment of ESC. Zhang and co-workers recommended that esophagectomy with extended lymphadenectomy should be considered as the primary treatment of choice for the early-stage ESC (8). To further clarify the efficacy of surgery, our study analyzed the surgical technique of patients and found that there was no significant difference in survival among patients undergoing McKeown, Ivor-Lewis, and Sweet esophagectomy. Moreover, less than half of the patients who underwent surgery received adjuvant treatment, including chemotherapy, radiotherapy, and chemoradiotherapy, and no patient received neoadjuvant treatment. Statistical analyses showed that adjuvant therapy did not prolong the survival of ESC patients. There is indeed a lack of evidence on the effectiveness of postoperative adjuvant therapy in patients with ESC, but few studies found that neoadjuvant radiotherapy or chemoradiotherapy might be effective (17–19). Future research on the therapeutic efficacy of ESC patients, especially prospective research and clinical trials in this area, needs to be further advanced.

Several studies have reported a favorable long-term outcome of ESC. Li and co-workers revealed that the 1-, 3-, and 5-year cancer-specific survival rates of ESC patients with esophagectomy were 79.1%, 61.3%, and 55.5%, respectively (20). Another small sample analysis of 24 patients with stages I–IV ESC further disclosed a 3- and 5-year survival rate of 83.3% and 70.8%, respectively (21). Despite existing prognostic data, comprehensive characterization remains lacking. We performed distinct PFS and OS analysis per stage, demonstrating a significant difference. Considering that the carcinomatous components of ESC are mostly SCC, we further compared the survival outcome of ESC with ESCC and showed that ESC patients had statistically better prognosis than did ESCC patients. This is consistent with most research findings. Zhang et al. thought that the longer 5-year survival seen in ESC patients lies in the fact that more patients in the ESC group were in earlier stages (8). Iyomasa et al. speculated that the difference in radical resection rate between ESC and ESCC may lead to a higher 3-year survival rate for ESC (9). Conversely, some reports indicated no difference in prognosis between ESC and ESCC, and even for T1 stage patients, the prognosis of ESC is worse (10, 20), yet we ensured reliability through PSM, minimizing confounding factors and setting an equivalent radical resection rate and pTNM stage distribution between the two groups. This provides reliability for our results to some extent and, on the other hand, further confirms that ESC and ESCC should not be synchronously treated as a singular disorder.

Accurate diagnosis of ESC is the primary prerequisite for effectively recognizing the disease and implementing precision treatment. However, due to its unique pathology, ESC is prone to misdiagnosis, as shown by our finding of 62.7% of patients not obtaining pathological confirmation until surgery was completed. It can be speculated that, at present, a portion of unresectable ESC patients may be misdiagnosed as ESCC. Kubo et al. reported a 71-year-old ESC patient who was almost misdiagnosed as ESCC, once again confirming the importance of improving biopsy accuracy or developing new diagnostic methods for ESC (5). We found significant differences in CT images between ESC and ESCC, consistent with the previously reported gross appearance of ESC (4). This provides a new approach for discriminating ESC from ESCC. Utilizing radiomics, we developed an objective prediction model containing one GLDM and one GLCM texture feature. Gldm_DependenceEntropy reflects intratumor heterogeneity via grayscale arrangement since entropy is inversely proportional to uniformity, representing the irregularity of image pixel intensity values (22). Glcm_ InformationalMeausureofCorrelation1 is related to the joint probability occurrence of the pixel pairs entropy (23). The lower the Glcm_ InformationalMeausureofCorrelation1 value, the less homogeneous the distribution of the intensities. Our data revealed an elevated Rad-score for ESC compared to ESCC, signifying the model’s efficacy and indirectly suggesting prevalent intratumoral heterogeneity in ESC. In addition, the AUCs, sensitivity, specificity, and calibration curve in both the training and validation cohorts confirmed the good performance of the prediction model.

Certain limitations exist in this study. Firstly, the retrospective nature of this analysis omitted certain patient information, like pathological data and treatment efficacy, crucial for further analysis. Secondly, the incorporation of advanced ESC patients into the radiomics model created mild constraints regarding its utilization.

## 5 Conclusions

This study focused extensively on the clinicopathological characteristics, prognosis, and radiomics features of the rare malignancy. ESC has a better prognosis and differs in CT images when compared with ESCC. By developing a radiomics prediction model, we provide reliability and convenience for the differential diagnosis of ESC from ESCC.

## Data availability statement

The raw data supporting the conclusions of this article will be made available by the authors, without undue reservation.

## Ethics statement

The studies involving humans were approved by Institutional Review Board (IRB) of West China Hospital. The studies were conducted in accordance with the local legislation and institutional requirements. The ethics committee/institutional review board waived the requirement of written informed consent for participation from the participants or the participants’ legal guardians/next of kin because the medical records or biological specimens used in this study were obtained from previous clinical diagnosis and treatment, and the risk to the subjects is no greater than minimal risk. Waiving informed consent will not have a negative impact on the rights and health of the subjects, and the privacy and personal identity information of the subjects will be protected. Written informed consent was not obtained from the individual(s) for the publication of any potentially identifiable images or data included in this article because the images used in this study were obtained from previous clinical diagnosis and treatment, and the risk to the subjects is no greater than minimal risk. Waiving informed consent will not have a negative impact on the rights and health of the subjects, and the privacy and personal identity information of the subjects will be protected.

## Author contributions

BH: Data curation, Formal analysis, Funding acquisition, Methodology, Writing – original draft. KZ: Conceptualization, Validation, Writing – original draft. YY: Data curation, Investigation, Resources, Writing – original draft. YZ: Formal analysis, Software, Writing – original draft. GL: Conceptualization, Investigation, Validation, Writing – review & editing. HZ: Supervision, Validation, Writing – review & editing. BZ: Conceptualization, Funding acquisition, Supervision, Validation, Writing – review & editing.
