# Supplementary material for: Investigating esophageal sarcomatoid carcinoma and its comparison with esophageal squamous cell carcinoma on clinicopathological characteristics, prognosis, and radiomics features: a retrospective study
Source: Front Oncol. 2024 Jul 1;14:1398982. doi: 10.3389/fonc.2024.1398982 (PMC11247005; doi:10.3389/fonc.2024.1398982)
Supplement: Supplementary file 1 [file DataSheet_1.docx]

Supplementary Material

# Supplementary Figures and Tables

## Supplementary Figures


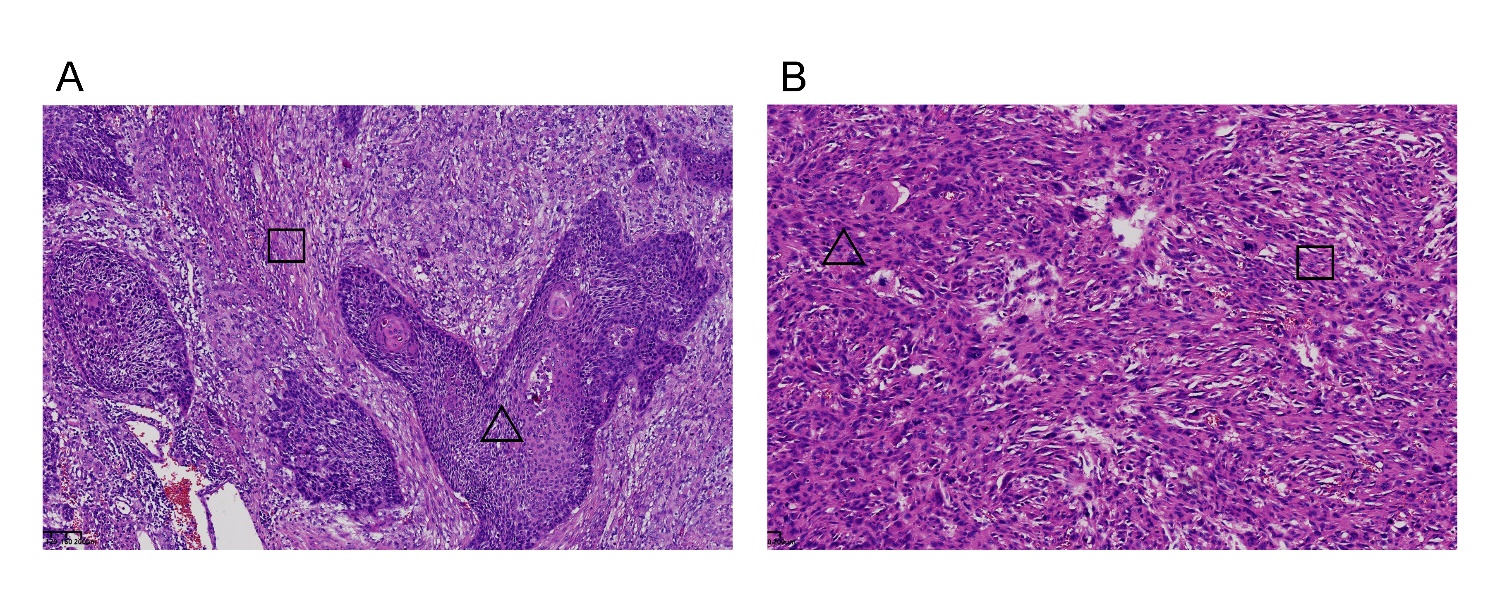


**Supplementary Figure 1** HE pictures of the surgically resected tumors with different proportion of carcinomatous and sarcomatous components. (A) The ×100 image shows a mass of epithelioid cancer cells (△) admix with few sarcomatoid spindle cells (□) from a 67-year-old male who was initially diagnosed as ESCC through endoscopic biopsy. (B) The ×100 image shows a large number of sarcomatoid spindle cells (□) with a small number of epithelioid cancer cells (△) from a 63-year-old male who was accurately diagnosed as ESC through endoscopic biopsy. ESCC, esophageal squamous cell carcinoma; ESC, esophageal sarcomatoid carcinoma.


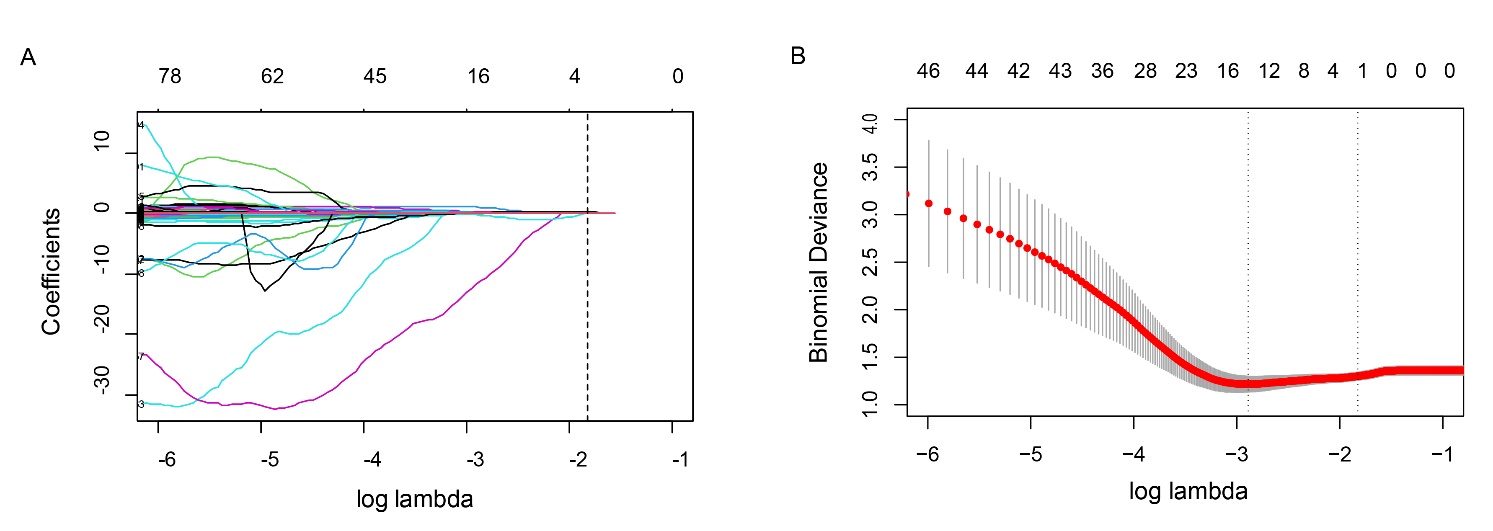


**Supplementary Figure 2** Selection of radiomics features for the differential diagnosis of ESC from ESCC using the LASSO-logistic regression model. (A) Coefficient profiles of radiomicis features. (B) The cross‑validation curve. Selection of the tuning parameter (lambda) was based on 10-fold cross-validation with the minimum criteria. LASSO, least absolute shrinkage and selection operator.

## Supplementary Tables

**Supplementary Table 1** Clinical characteristics of patients with non-resected ESC.

| Variables | Overall (n=8) | Variables | Overall (n=8) |
| --- | --- | --- | --- |
| Age (mean ± SD) | 63.3 ± 13.3 | T stage (%) |  |
| Age, years (%) |  | T1 | 0 (0.0) |
| ≤ 60 | 3 (37.5) | T2 | 0 (0.0) |
| > 60 | 5 (62.5) | T3 | 1 (12.5) |
| Gender (%) |  | T4 | 1 (12.5) |
| Male | 8 (100.0) | Unknown | 6 (75.0) |
| Female | 0 (0.0) | N stage (%) |  |
| Tobacco (%) |  | N0 | 0 (0.0) |
| Yes | 6 (75.0) | N1 | 2 (25.0) |
| No | 2 (25.0) | N2 | 0 (0.0) |
| Alcohol (%) |  | N3 | 0 (0.0) |
| Yes | 7 (87.5) | Unknown | 6 (75.0) |
| No | 1 (12.5) | Treatment (%) |  |
| Tumor location (%) |  | Palliative surgery | 1 (12.5) |
| Upper thoracic | 2 (25.0) | Chemoradiotherapy | 3 (37.5) |
| Middle thoracic | 4 (50.0) | Chemotherapy | 2 (25.0) |
| Lower thoracic | 2 (25.0) | None | 2 (25.0) |
| Histological grade (%) |  |  |  |
| G1 | 0 (0.0) |  |  |
| G2 | 0 (0.0) |  |  |
| G3 | 5 (62.5) |  |  |
| Gx | 3 (37.5) |  |  |

ESC, esophageal sarcomatoid carcinoma; SD, standard deviation.

**Supplementary Table 2** Clinical characteristics of resectable ESC and ESCC patients before and after PSM.

| Variables | Before PSM | | | After PSM | | |
| --- | --- | --- | --- | --- | --- | --- |
|  | [Sarcomatoid](javascript:;) [carcinoma](javascript:;)  (n=59) | Squamous cell carcinoma  (n=2856) | *p* value | [Sarcomatoid](javascript:;) [carcinoma](javascript:;)  (n=51) | Squamous cell carcinoma  (n=98) | *p* value |
| Age (mean ± SD) | 62.4 ± 8.6 | 60.7 ± 8.0 |  | 62.9 ± 8.5 | 60.9 ± 8.5 |  |
| Age, years (%) |  |  | 0.598 |  |  | 0.488 |
| ≤ 60 | 25 (42.4) | 1330 (46.6) |  | 20 (39.2) | 45 (45.9) |  |
| > 60 | 34 (57.6) | 1526 (53.4) |  | 31 (60.8) | 53 (54.1) |  |
| Gender (%) |  |  | 0.252 |  |  | 0.792 |
| Male | 51 (86.4) | 2287 (80.1) |  | 44 (86.3) | 87 (88.8) |  |
| Female | 8 (13.6) | 569 (19.9) |  | 7 (13.7) | 11 (11.2) |  |
| Tumor location (%) |  |  | 0.564 |  |  | 0.446 |
| Upper thoracic | 5 (8.5) | 293 (10.2) |  | 5 (9.8) | 6 (6.1) |  |
| Middle thoracic | 41 (69.5) | 1795 (62.9) |  | 38 (74.5) | 69 (70.4) |  |
| Lower thoracic | 13 (22.0) | 768 (26.9) |  | 8 (15.7) | 23 (13.5) |  |
| Histological grade (%) |  |  | < 0.001 |  |  | 0.870 |
| G1 | 1 (1.7) | 31 (1.1) |  | 14 (27.5) | 22 (22.5) |  |
| G2 | 3 (5.1) | 1343 (47.0) |  | 1 (2.0) | 2 (2.0) |  |
| G3 | 34 (57.6) | 1375 (48.1) |  | 3 (5.9) | 5 (5.1) |  |
| Gx | 21 (35.6) | 107 (3.7) |  | 33 (64.6) | 69 (70.4) |  |
| pT stage (%) |  |  | 0.028 |  |  | 0.691 |
| T1 | 17 (28.8) | 566 (19.8) |  | 17 (33.3) | 24 (24.5) |  |
| T2 | 16 (27.1) | 487 (17.1) |  | 12 (23.6) | 26 (26.5) |  |
| T3 | 21 (35.6) | 1406 (49.2) |  | 17 (33.3) | 34 (34.7) |  |
| T4 | 5 (8.5) | 397 (13.9) |  | 5 (9.8) | 14 (14.3) |  |
| pN stage (%) |  |  | 0.289 |  |  | 0.878 |
| N0 | 38 (64.4) | 1482 (51.9) |  | 33 (64.7) | 61 (62.2) |  |
| N1 | 12 (20.3) | 821 (28.7) |  | 10 (19.6) | 18 (18.4) |  |
| N2 | 8 (13.6) | 421 (14.7) |  | 8 (15.7) | 19 (19.4) |  |
| N3 | 1 (1.7) | 132 (4.6) |  | 0 (0.0) | 0 (0.0) |  |
| pTNM stage (%) |  |  | < 0.001 |  |  | 0.773 |
| I | 16 (27.1) | 483 (16.9) |  | 12 (23.5) | 19 (19.4) |  |
| II | 28 (47.5) | 921 (32.2) |  | 25 (49.0) | 44 (44.9) |  |
| III | 14 (23.7) | 1135 (39.7) |  | 14 (27.5) | 34 (34.7) |  |
| IV | 1 (1.7) | 317 (11.1) |  | 0 (0.0) | 1 (1.0) |  |

ESC, esophageal sarcomatoid carcinoma; ESCC, esophageal squamous cell carcinoma; PSM, propensity score matching; SD, standard deviation; pT, pathological T; pN, pathological N; pTNM, pathological TNM.

**Supplementary Table 3** Comparison of patients’ characteristics between the training cohort and validation cohort.

| Variables | Training cohort (n=82) | Validation cohort (n=27) | *p* value |
| --- | --- | --- | --- |
| Age (mean ± SD) | 62.3 ± 8.7 | 61.9 ± 6.3 |  |
| Age, years (%) |  |  | 0.500 |
| ≤ 60 | 33 (40.2) | 12 (48.1) |  |
| > 60 | 49 (59.8) | 15 (51.9) |  |
| Gender (%) |  |  | 0.822 |
| Male | 75 (91.5) | 23 (44.4) |  |
| Female | 7 (8.5) | 4 (55.6) |  |
| Tumor location (%) |  |  | 1.000 |
| Upper thoracic | 6 (7.3) | 2(7.4) |  |
| Middle thoracic | 60 (73.2) | 20 (74.1) |  |
| Lower thoracic | 16 (19.5) | 5 (18.5) |  |
| Histological type (%) |  |  |  |
| ESC | 50 (61.0) | 15 (55.6) | 0.656 |
| ESCC | 32 (39.0) | 12 (44.4) |  |
| Histological grade (%) |  |  | 0.598 |
| G1 | 2 (2.4) | 0 (0.0) |  |
| G2 | 4 (4.9) | 3 (11.1) |  |
| G3 | 58 (70.7) | 18 (66.7) |  |
| Gx | 18 (22.0) | 6 (22.2) |  |
| pT stage (%) |  |  | 0.558 |
| T1 | 12 (14.6) | 7 (25.9) |  |
| T2 | 20 (24.4) | 7 (25.9) |  |
| T3 | 36 (43.9) | 10 (37.1) |  |
| T4 | 14 (17.1) | 3 (11.1) |  |
| pN stage (%) |  |  | 0.385 |
| N0 | 49 (59.8) | 13 (48.1) |  |
| N1 | 14 (17.1) | 8 (29.7) |  |
| N2 | 19 (23.1) | 6 (22.2) |  |
| N3 | 0 (0.0) | 0 (0.0) |  |
| pTNM stage (%) |  |  | 0.776 |
| I | 9 (11.0) | 4 (14.8) |  |
| II | 40 (48.8) | 11 (40.8) |  |
| III | 32 (39.0) | 12 (44.4) |  |
| IV | 1 (1.2) | 0 (0.0) |  |

SD, standard deviation; ESC, esophageal sarcomatoid carcinoma; ESCC, esophageal squamous cell carcinoma; pT, pathological T; pN, pathological N; pTNM, pathological TNM.
